# Oxygen-saturation-related functional parameter as a biomarker for diabetes mellitus—extraction method and clinical validation

**DOI:** 10.3389/fcell.2023.1195873

**Published:** 2023-05-11

**Authors:** Jinze Zhang, Zhongzhou Luo, Gengyuan Wang, Yuancong Huang, Keyi Fei, Yushuang Liu, Jiaxiong Li, Jin Yuan, Peng Xiao

**Affiliations:** State Key Laboratory of Ophthalmology, Zhongshan Ophthalmic Center, Sun Yat-sen University, Guangdong Provincial Key Laboratory of Ophthalmology and Visual Science, Guangdong Provincial Clinical Research Center for Ocular Diseases, Guangzhou, China

**Keywords:** fundus photography, diabetes mellitus, retina vessel segmentation, oxygen saturation level, light reflection analysis

## Abstract

**Purpose:** To develop a computational method for oxygen-saturation-related functional parameter analysis of retinal vessels based on traditional color fundus photography, and to explore their characteristic alterations in type 2 diabetes mellitus (DM).

**Methods:** 50 type 2 DM patients with no-clinically detectable retinopathy (NDR) and 50 healthy subjects were enrolled in the study. An optical density ratio (ODR) extraction algorithm based on the separation of oxygen-sensitive and oxygen-insensitive channels in color fundus photography was proposed. With precise vascular network segmentation and arteriovenous labeling, ODRs were acquired from different vascular subgroups, and the global ODR variability (ODR_v_) was calculated. Student’s t-test was used to analyze the differences of the functional parameters between groups, and regression analysis and receiver operating characteristic (ROC) curves were used to explore the discrimination efficiency of DM patients from healthy subjects based on these functional parameters.

**Results:** There was no significant difference in the baseline characteristics between the NDR and healthy normal groups. The ODRs of all vascular subgroups except the micro venule were significantly higher ( p<0.05 , respectively) while ODR_v_ was significantly lower ( p<0.001 ) in NDR group than that in healthy normal group. In the regression analysis, the increased ODRs except micro venule and decreased ODR_v_ were significantly correlated with the incidence of DM, and the C-statistic for discrimination DM with all ODR is 0.777 (95% CI 0.687-0.867, p<0.001 ).

**Conclusion:** A computational method to extract the retinal vascular oxygen-saturation-related optical density ratios (ODRs) with single color fundus photography was developed, and increased ODRs and decreased ODR_v_ of retinal vessels could be new potential image biomarkers of DM.

## 1 Introduction

The eye is a structurally advanced optical organ with a complex vascular network. The maintenance of retinal function is critically dependent on the normal functioning of the blood-retinal barrier (Kaur et al., 2008; O’Leary and Campbell, 2023). Fundus diseases and related systematic diseases can cause functional changes in the fundus such as ischemia and hypoxia, which can damage the blood-retinal barrier, leading to retinal vascular permeability changes, hemorrhage, exudation, neovascularization, and other lesions (Pournaras et al., 2008; Ascunce et al., 2023; Monickaraj et al., 2023). Diabetes mellitus (DM) is a common endocrine metabolic disease with its microvascular complications in the eye that can result in diabetic retinopathy (DR), which is the leading cause of blindness in working-age people (Bourne et al., 2021; Steinmetz et al., 2021). Hypoxia is considered to be the core pathogenesis of DR besides of hyperglycemia in retinal blood vessels caused by DM (Hardarson, 2013). Hypoxia along with hyperglycemia stimulate vascular endothelium and eventually lead to vascular endothelial cell dysfunction, which in turn leads to the occurrence of DR. (Hardarson and Stefánsson, 2012; Khoobehi et al., 2013). Therefore, it is equally important to detect retinal oxygen saturation as well as blood glucose in DM patients, which can help to prompt the potential occurrence of DR and avoid further visual impairment.

Retinal vessel is the only vascular system in the body that can be observed *in vivo* (Hanssen et al., 2022), and non-invasive imaging technique is the first choice for retinal vascular oxygen saturation measurement. Nevertheless, there are only limited imaging instruments available to measure retinal oxygen saturation, in which dual-wavelength retinal oximetry is a promising technique. By measuring the light of different wavelengths reflected from the eye, it takes advantage of the light absorption variations between oxyhemoglobin and hemoglobin, and a calculation can be made that correlates directly to the retinal vascular oxygenation (Beach, 2014; Jeppesen and Bek, 2019; Garg et al., 2021). Previous studies using retinal oximetry have found that the oxygen saturation of retinal vessels in DR is significantly higher than that in normal subjects, and increases with the severity of DR (Hardarson and Stefánsson, 2012; Khoobehi et al., 2013; Jørgensen and Bek, 2014). Retinal oximetry has also been used to monitor retinal oxygen saturation in other eye diseases such as central retinal vein occlusion (CRVO) and glaucoma, showing varying degrees of increase in retinal oxygen saturation (Stefánsson et al., 2019). However, despite its potential, retinal oximetry is not widely used in ophthalmology clinics due to its recent commercialization.

Traditional color fundus cameras are the most commonly used equipment for fundus examination and disease screening (Karlsson et al., 2021). Their broad-spectrum cameras use complementary metal-oxide semiconductor (CMOS) or charge couple device (CCD) photoreceptors covered with red-green-blue (RGB) filters (Malvar et al., 2004). Thus, oxygen-sensitive and insensitive wavelengths reflected from retina vessels, which corresponds to different oxyhemoglobin and hemoglobin light absorptions, are generally received in the red and green channels, respectively, allowing for the estimation of blood oxygen function by extracting light absorption information from these channels.

In this study, we aim to develop a computational method for analyzing oxygen-saturation-related functional parameters with traditional color fundus photography based on precise vessel segmentation and light absorption analysis, providing an easy-to-apply image analysis algorithm for traditional color fundus cameras. With that, we also explore the characteristic alterations of retinal vascular oxygen function in type 2 DM patients to look for new potential image biomarkers for the monitoring of DM.

## 2 Methods

### 2.1 Ethics

This was a retrospective observational study. The project underwent a formal administrative review and was determined to be methodologically validated according to the institutional policy of Zhongshan Ophthalmic Center, Sun Yat-sen University. Therefore, this study was considered not to be a human subject’s study, but the study was still reviewed by Institutional Review Board to avoid information leakage during data processing (protocol number: 2017KYPJ104).

### 2.2 Population

We included 50 patients with type 2 diabetes mellitus (DM) and a normal group of 50 age- and sex-matched healthy normal subjects between 1 January 2019 and 15 May 2021. All the subjects were adults. We extracted the age and sex information of the subjects from the medical records for intergroup matching and determined whether the subjects are patients with type 2 DM or not. To assess the existence of diabetic retinopathy, standard seven-fields fundus photographs were obtained with a mydriasis-free digital fundus camera (RetiCam 3100, SYSEYE, China). Patients with clinically detectable retinopathy, or with a history of ocular disease, inflammation, trauma, or any intraocular surgery were excluded. Further exclusion criteria include related systemic diseases, such as Alzheimer’s disease, hypertension or lung disease. The healthy normal group should also meet the above exclusion criteria in addition to no diabetes.

### 2.3 Vessel segmentation and labeling

**FIGURE 1:**
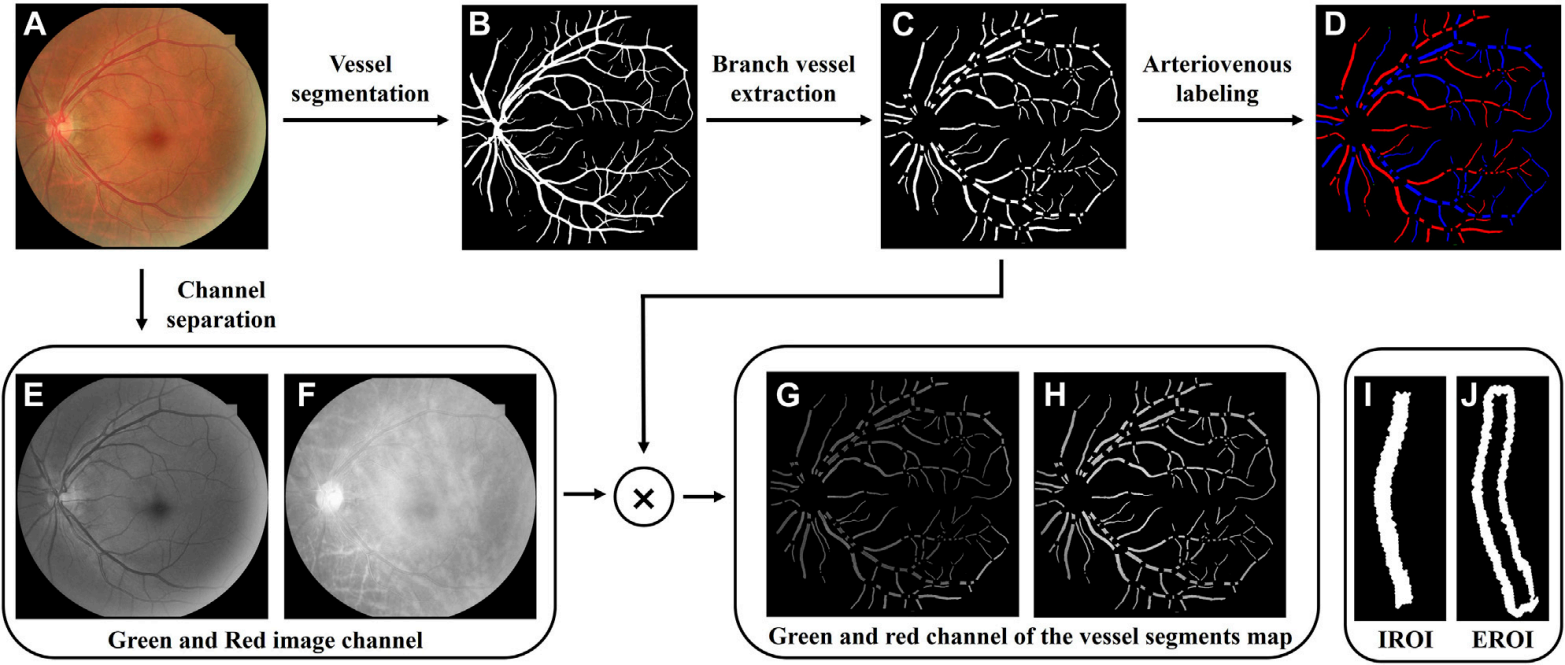
Region of interest extraction process. **(A)** Original color fundus photography. **(B)** Retinal vascular binarization network. **(C)** Retinal vessel segments map. **(D)** Arterial and venous labeling. **(E,F)** Green and red image channels extracted from the original fundus image. **(G,H)** Green and red channel of the vessel segments map. **(I,J)** Intravascular region of interest (IROI) and extravascular region of interest (EROI).

Only the 50° fundus photography centered on the macula are analyzed. Figure 1 shows the flowchart for the processing of the region of interest (ROI) of retinal vessels. To extract the retinal vascular network, we employed an intelligent automatic vascular segmentation method, details of which are available in our earlier publication (Wang et al., 2021; 2023). Briefly, the original fundus color photographs (Figure 1A) were processed with an enhanced U-net by employing a multipath attention network model (MA-net) for improved vessel segmentation, achieving an area under the curve (AUC) of 0.9838 for segment accuracy (Wang et al., 2023). Blood vessel junctions on the obtained retinal vascular binarization network (Figure 1B) were detected then through a convolution method on vessel skeleton, facilitating the vessel segments extraction (Figure 1C). The arteriovenous labeling of the segmented vessels was further processed based on an intra-image regularization classifier (Figure 1D) (Xu et al., 2017).

To ensure the calculation of the oxygen-saturated-related functional parameter of retinal vessels, the red and green channels (Figures 1E,F) composing the original fundus color photography were extracted through channel separation. By multiplying them with the binarized vessel segments map, oxygen-sensitive and insensitive wavelengths reflected from the retinal vascular areas were obtained (Figures 1G,H), of which each vessel segment was marked as the intravascular regions of interest (IROI, Figure 1I). To obtain the light reflection information of the retinal background around the vessel segment, a range of one vessel diameter is extended outward along the blood vessel segment outlines, which was marked as the extravascular region of interest (EROI, Figure 1J).

### 2.4 Optical density ratio extraction

According to the EROI and IROI of blood vessels, the optical density (OD) values were calculated for every three pixels along the vascular central line (Formula 1), the red channel is used as the oxygen sensitive channel, and the green channel represents the oxygen insensitive channel, and the optical density ratio (ODR) of the two is defined as the blood oxygen-saturation-related parameter (Formula 2) (Tiedeman et al., 1998; Beach et al., 1999).

$$\begin{array}{c} OD=\log_{10}\frac{O_{EROI}}{O_{IROI}} \end{array}$$ (1)

$$\begin{array}{c} ODR=\frac{{OD}_{red}}{{OD}_{green}} \end{array}$$ (2)

In order to study the differences of different types of blood vessels, all branch vessels output ODR according to the labeling of arteries and veins, and distinguish between main and micro vessels referring to the setting of commercial retinal oximeter (Stefánsson et al., 2017), i.e., ≥6pixel diameter (1pixel≈12.69 μm in this study) was defined as main vessels and <6pixel diameter was defined as micro vessels. Finally, ODR outputs the results according to all vessels (All), all arteriole (A), main arteriole (main A), micro arteriole (mic A), all venule (V), main venule (main V), and micro venule (mic V).

In addition to the ODR of each vascular subgroup, we also average the absolute value of the difference of ODR between all adjacent blocks of pixels and define it as ODR variability (ODR_v_, Formula 3).

$$\begin{array}{c} {ODR}_{v}=\frac{\sum \left|{ODR}_{n}-{ODR}_{n-1}\right|}{n-1} \end{array}$$ (3)

### 2.5 Statistical analysis

Student’s t-test or chi-square test used to compare the differences between Normal and DM groups. All data were recorded as mean ± standard deviation (SD). Data distribution was analyzed by the Kolmogorov–Smirnov test to determine normally distributed data ( P > 0.05 ). Logistics regression was used to analyze the relationship between ODR and DM in each vascular subgroup and to generate ROC curve to evaluate the diagnostic efficiency of retinal vascular function parameters. All statistical analyses were performed using SPSS software (25.0; IBM Corporation, Armonk, NY). GraphPad Prism 9.0 was used for data visualization.

## 3 Results

There is no difference in the age and sex between the 50 healthy volunteers in Normal group and the 50 patients in DM group (Table 1).

**TABLE 1:** Clinical parameters and ODR of study subjects.

	Normal	DM	T-statistic	*p* value
No, of subject	50	50		
Age, yrs	43.4 (4.5)	43.9 (4.8)	−0.555	0.58
Sex, female, no (%)	23 (46%)	24 (48%)	NA	0.55
Duration of diabetes, yrs	/	4.16 (4.03)		
Fasting plasma glucose, mmol/L	/	8.89 (4.55)		
Optical density ratio, ODR				
All vessels	0.711 (0.060)	0.754 (0.082)	−3.037	0.03
All arteriole	0.713 (0.057)	0.755 (0.083)	−2.997	0.04
All venule	0.709 (0.063)	0.754 (0.082)	−3.037	0.03
Main arteriole	0.728 (0.056)	0.773 (0.083)	−3.199	0.02
Main venule	0.717 (0.058)	0.761 (0.082)	−3.092	0.03
Micro arteriole	0.634 (0.068)	0.672 (0.096)	−2.263	0.026
Micro venule	0.599 (0.082)	0.627 (0.117)	−1.405	0.164
Variability (ODR_v_)	0.273 (0.025)	0.236 (0.059)	3.915	<0.001

a. Student’s t-test or chi-square test used to compare difference between groups.

Table 1 alsodemonstrates the ODR for all vascular subgroups, ODR_v_, as well as the t-statistic and *p*-values. In both healthy normal group and DM group, A ODR is higher than V ODR (mean ± SD, A vs. V, Normal: 0.713 ± 0.057 vs. 0.708 ± 0.063; DM: 0.755 ± 0.083 vs. 0.754 ± 0.082), main A ODR is higher than main V ODR (Normal: 0.728 ± 0.056 vs. 0.717 ± 0.058; DM: 0.773 ± 0.083 vs. 0.761 ± 0.082) and micro A is higher than micro V ODR (Normal: 0.634 ± 0.068 vs. 0.599 ± 0.082; DM: 0.672 ± 0.096 vs. 0.627 ± 0.117), which means that ODR is positively correlated with blood oxygen saturation. Compared to Normal group, ODR is significantly increased in all vascular subgroups except micro venular in DM group ( p<0.05 , respectively). ODR_v_ is significantly decreased in DM group ( p<0.001 ).

**FIGURE 2:**
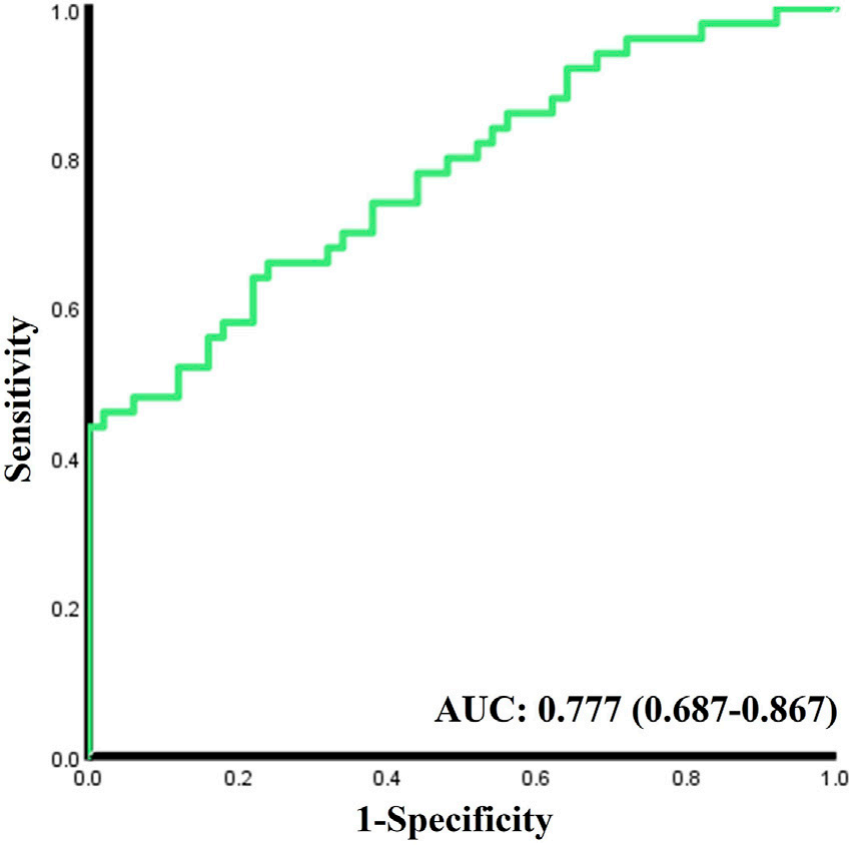
ROC curves of predictive discrimination for DM.

As shown in the logistics regression results in Table 2, higher ODRs are significantly associated with the occurrence of DM, especially in main A (OR 1.953, 95% CI 1.252-3.046) and main V (OR 1.908, 95% CI 1.227-2.968), and also in micro A (OR 1.604, 1.047-2.455). While Lower ODR_v_ (OR 0.381, 95% CI 0.210-0.693) is significantly associated with the occurrence of DM. The C-statistic for the discrimination of DM from the healthy normal group combining all the acquired oxygen-saturation-related functional parameters is 0.777 (95% CI 0.687-0.867, p<0.001 ) (Figure 2).

**TABLE 2:** Variables in the equation of the logistics regression.

	OR	95% CI		*p* value
		Lower	Upper	
All vessels	1.885	1.214	2.925	0.005
All arteriole	1.863	1.201	2.891	0.006
All venule	1.884	1.215	2.923	0.005
Main arteriole	1.953	1.252	3.046	0.020
Main venule	1.908	1.227	2.968	0.004
Micro arteriole	1.604	1.047	2.455	0.030
Micro venule	1.334	0.889	2.002	0.166
Variability	0.381	0.210	0.693	0.002

a. Logical regression was used to analyze the relationship between ODR, and DM, in each vascular subgroup and variability.

## 4 Discussion

In this study, we present a computational approach for extracting oxygen-saturation-related functional parameters through precise vascular segmentation and light reflection channel analysis using single traditional color fundus photography. By applying this method to the comprehensive analysis of the fundus images acquired from normal individuals and patients with type 2 diabetes, we revealed that the retinal vascular ODRs were increased while the ODR variability was reduced significantly in DM patients, providing new potential image biomarkers for the management of DM.

The successful extraction of retinal vascular ODRs with color fundus photography offers a new protocol for retinal vascular function evaluation, providing additional quantitative functional information based on the most commonly used fundus imaging tools in clinical settings. Compared to the current existed retinal oximetry, which only reveals oxygen saturation level in main retinal vessels and requires subjects to withstand long light exposure time (Hardarson, 2013), our method achieves information in microvascular level without any additional hardware implementation, which ensures its convenience and accessibility with greatly reduced cost. These advantages suggest extensive potential application value in the diagnosis and screening of retinal diseases.

In our study, we have shown that the increase of proposed oxygen-saturation-related ODRs were statistically significant at the stage of DM, which could be related to the stronger affinity of glycosylated hemoglobin to oxygen in DM (Graham et al., 1980; van Kampen and Zijlstra, 1983). Indeed, former studies using retinal oximetry have found obvious increase in retinal oxygen saturation level during DR, especially in proliferative DR, but no significant change was shown in DM stage before (Khoobehi et al., 2013). This indicates that our newly proposed ODRs might be more sensitive parameters than that of commercial retinal oximetry in finding alterations in blood oxygen function. Moreover, our exploration of ODR in the microvascular groups revealing significant increase in micro arterioles of DM patients further proves the high sensitivity of our method.

We have proposed and analyzed the ODR variability for the first time on the basis of extracted ODR of the full retinal vascular network and found it to be significantly decreased in DM patients compared to normal subjects. While glycosylated hemoglobin has higher affinity to oxygen (Graham et al., 1980), it damps the oxygen-release capacity (Ditzel, 1972) of retinal blood, results in reduced oxygen saturation variations. Studies have also found that the retinal blood flow velocity increases in DM patients (Landa et al., 2011), meaning less oxygen release time of blood in retinal vessels, further reduced the ODR variability.

Our method suffers limited retinal vessel resolution and imaging depth due to the physical nature of fundus photography. Only the superficial retinal vessels can be analyzed without 3-dimensional (3D) illustration. The same limitations also exist in current commercial retinal oximetry. Recently, visible-light optical coherence tomography based techniques for retinal oxygen saturation measurement have been gradually proposed (Pi et al., 2020; Poddar and Basu, 2020), achieving high resolution 3D imaging of retinal vascular oxygen function information (Pi et al., 2020), although most of these studies are still in laboratorial stage.

Our attempt to distinguish DM from normal subjects with all the extracted functional ODR parameters showed a relative low C statistic result (AUC = 0.777), which is not sufficient for DM diagnosis or screening. Since our previous study found that the morphological parameters of retinal vessels in DM patients also changed significantly (Li et al., 2021), further combining functional and morphological parameters of retinal blood vessels and multi-center studies or follow-up studies may help us better understand the changes in retinal blood vessel characteristics during the occurrence and development of DM and even DR, so as to diagnose DM or DR More accurately. Furthermore, applying our method to other eye diseases related to retinal vascular oxygen function alterations such as glaucoma (Vandewalle et al., 2014), central retinal vein occlusion (Hardarson and Stefánsson, 2010), age-related macular degeneration (Geirsdottir et al., 2014), etc. in the future would further demonstrate the reliability and universality of our method.

## Data Availability

The raw data supporting the conclusions of this article are available from the corresponding authors upon reasonable request.
